# Intraoperative Navigation in Cervical Spine Surgery

**DOI:** 10.3390/jcm15051746

**Published:** 2026-02-25

**Authors:** Ahmed Majid Heydar, Masato Tanaka, Said Erkam Baykan, Mehmet Burak Yalçın, Uğur Özdemir, Abdülhalim Akar, Serdar Şirazi, Mustafa Kürklü

**Affiliations:** 1 Orthopedic and Traumatology Clinic, Memorial Bahçelievler Hospital, Bahçelievler Merkez, Adnan Kahveci Blv. No 227, 34180 Bahçelievler, Istanbul, Turkey; 2 Department of Orthopedics and Rehabilitation, Faculty of Medicine, Istanbul AREL University, Merkez Efendi Mahallesi, Eski Londra Asfaltı Cd., No 1/3, 34010 Zeytinburnu, Istanbul, Turkey; 3 Department of Orthopedic Surgery, Okayama Rosai Hospital, 1-10-25 Chikkomidorimachi, Okayama 702-8055, Japan; 4 Department of Orthopedics and Traumatology, Faculty of Medicine, Bandırma Onyedi Eylül University, Yeni, 10250 Bandırma, Balıkesir, Turkey; 5 Department of Orthopedic and Traumatology, Faculty of Medicine, Haliç University, 5. Levent Mh. 15 Temmuz Şehitler Cd., 34060 Eyüpsultan, Istanbul, Turkey; 6 Independent Researcher, Florya Plaza, Şenlikköy Mah., Eski Halkalı Cad., No 3, 34153 Florya, Istanbul, Turkey

**Keywords:** intraoperative navigation, cervical spine, pedicle screw, decompression, minimally invasive

## Abstract

**Background/Objectives**: Intraoperative navigation is predominantly utilized in thoracolumbar spine surgeries; however, its application in cervical procedures has swiftly increased in prevalence. Despite the growing prevalence of these systems, there is a paucity of scholarly publications that address the historical development, delineate the fields of application, and discuss the benefits and drawbacks of this growingly prevalent technology in cervical spine surgery. Our aim was to provide a succinct summary of the history of cervical spine navigation systems, zones of implementation, associated advantages and disadvantages, and recommendations for future improvements. **Methods**: We conducted an extensive literature review focusing on the evolution and application of intraoperative navigation technology in cervical spine surgery. The research sources included peer-reviewed journals indexed in PubMed, data from clinical trials, and case studies that examined various navigation systems, with particular emphasis on the latest intraoperative navigation technologies. **Results**: In addition to facilitating minimally invasive approaches in cervical spine surgery, intraoperative navigation systems have been successfully employed in various decompression procedures, corpectomies, and tumor excisions. The accurate and safe placement of implants has been significantly enhanced in all cervical spine fixation techniques, particularly in those requiring high precision, such as occipital condyle, odontoid, transarticular, and translaminar screw fixations. However, technical difficulties, increased radiation exposure to patients, and high costs remain significant challenges that must be addressed. **Conclusions**: Intraoperative navigation systems in cervical spine surgery have demonstrated efficacy across various cervical spine procedures, offering additional advantages in facilitating minimally invasive approaches. However, the technical challenges associated with their use, which impact accuracy, as well as increased radiological exposure and cost, represent significant drawbacks that warrant attention in future research.

## 1. Introduction

Surgical navigation is the process of tracing patient images obtained preoperatively or intraoperatively into the surgical field using navigation software to orient the surgeon in regions with complex anatomy and to improve the accuracy of procedures. Technological progress has led to the development of advanced intraoperative CT-based navigation with robotic assistance [1]. In its most recent form, two phases of spinal imaging have been incorporated: preoperative and intraoperative [2]. The registration of these imaging data with intraoperative anatomy provides valuable real-time visual feedback; for example, the position of the placed implant can be visualized in the patient’s images [1,2]. Therefore, enhancing the safety of the spine surgeries by minimizing the complications associated with implant malposition [3], and ensuring adequate decompression with consequent improved clinical outcomes and reduced revision surgeries [4,5,6].

In spinal surgery, the precision of implant placement, including screws, is crucial, as inaccurate screw placement may lead to devastating vascular injuries or permanent neural impairment. This fact is even more important in cervical spine and cranio-vertebral junction surgery because of the close proximity of crucial structures and limited working space, in addition to relatively smaller bones for fixation [7]. Implant placement in the upper cervical spine is like playing in a minefield; minimal medial deviation from the optimum trajectory may lead to injury in the spinal cord, while slight lateral deviation may injure the vertebral artery, which are examples of surgical challenges and potential explanations for the high complication rate in cervical spine surgery [8]. Therefore, the utility of intraoperative navigation systems in cervical spine surgery can be summarized in terms of facilitating surgical procedures, improving accuracy, and minimizing invasiveness [9].

Despite the proposed benefits offered by intraoperative navigation systems to cervical spine surgery, concerns about the accuracy, financial cost, and steep learning curve were raised [10,11,12]. Screening literature demonstrates that fluoroscopy-guided cervical spine instrumentation is still the most popular guidance method adopted and published [3], and absence of the cervical spine focused navigation reviews that integrate decompression, fixation, MIS, and future technologies. Therefore, this narrative review intends to offer a brief summary of the history of navigation in the cervical spine, fields of utility, benefits, pitfalls, and expected innovations.

## 2. Materials and Methods

A comprehensive literature search was performed in PubMed database, concentrating on the evolution and application of intraoperative navigation technology in cervical spine surgery. To enhance transparency and reproducibility, a structured search strategy and explicit study selection process were applied, consistent with recommendations for narrative reviews published in the Journal of Clinical Medicine. Elements of the PRISMA framework were incorporated where appropriate, although no formal meta-analysis was performed. The search employed a combination of keywords and MeSH terms related to “intraoperative navigation,” “cervical spine,” “spinal surgery,” and “neuronavigation.” The search strategy was supplemented by manual screening of reference lists from relevant articles and reviews to identify additional pertinent studies. The search was focused on recent articles reported after 2000, when intraoperative navigation became popular and advancements occurred.

### 2.1. Study Selection

Titles and abstracts retrieved from the initial search were independently screened by three reviewers (S.E.B., M.B.Y and S.Ş) to identify potentially relevant studies. Full-text articles of selected abstracts were then assessed for eligibility. Discrepancies were resolved through discussion or consultation with a senior reviewer (M.T.).

### 2.2. Inclusion and Exclusion Criteria

Studies were included if they:

- Focused on intraoperative navigation techniques applied to cervical spine surgery.
- Reported on clinical outcomes, safety, accuracy, or procedural efficiency.
- Were original research articles, including randomized controlled trials, cohort studies, case series, and comparative studies (all designs were considered eligible).

Exclusion criteria were:

- Studies not involving cervical spine procedures.
- Technical notes, or biomechanical analyses without clinical data.
- Reviews, editorials, and conference abstracts without full data.
- Non-English publications.

### 2.3. Evidence Synthesis

**Figure 1:**
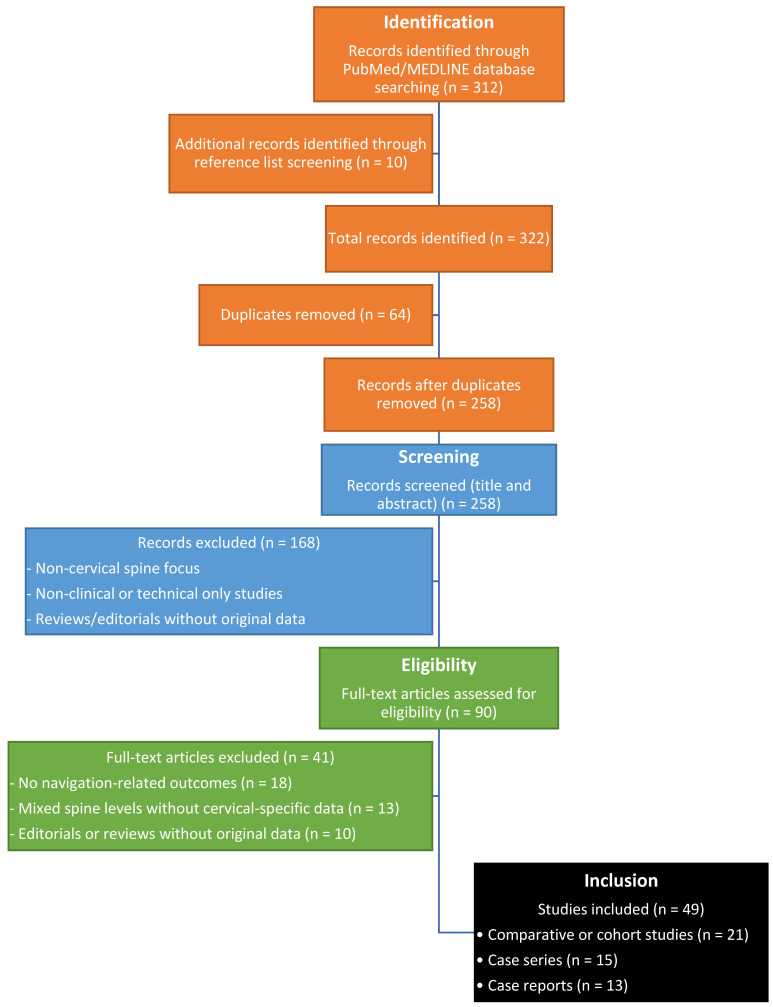
PRISMA-style flow diagram illustrating the identification, screening, eligibility assessment, and inclusion of studies.

Data from included studies were extracted systematically, focusing on study design, patient populations, navigation techniques, outcomes, and study quality. Given the narrative nature of this review, a qualitative synthesis was performed, emphasizing comparative analyses where available. A simple flow diagram (Figure 1) illustrates the selection process.

Due to heterogeneity in navigation systems, study designs, and outcome measures, quantitative synthesis was not feasible. Findings were therefore synthesized narratively.

Comparative and cohort studies were emphasized when discussing accuracy, safety, and clinical effectiveness, whereas case reports were used to illustrate rare anatomical scenarios, complex pathology, or uncommon complications and were not used to infer comparative effectiveness.

### 2.4. Study Quality and Risk of Bias Assessment

For key comparative studies (e.g., navigation vs. fluoroscopy), methodological quality was appraised using appropriate tools such as the Newcastle–Ottawa Scale for observational studies and the Cochrane Risk of Bias Tool for randomized trials. Each study was rated for potential bias in selection, measurement, and reporting.

## 3. Results

Valuable information was obtained from database screening regarding different aspects of intraoperative navigation in the cervical spine, and we attempted to summarize them under the following subtopics:

### 3.1. Historical Evolution

In cervical spine surgery, one of the primitive methods of image guidance in cervical spine surgery is the use of intraoperative lateral radiographs to ensure correct screw trajectory and minimize potential complications [13]. Nevertheless, the reliability of this method has been questioned in multiple studies, indicating that radiography has low accuracy in identifying pedicle screw misplacement [14]. The integration of fluoroscopy into spinal surgery marks a significant improvement in image-guided surgery. It provides numerous advantages over radiographic guidance, such as rapid multidirectional imaging. These benefits, along with the widespread accessibility of fluoroscopic units, have led to their extensive adoption. A global survey revealed that 78% of spine surgeons routinely use fluoroscopic navigation as their primary image-guidance technique [11]. The factors influencing hardware misplacement during fluoroscopic guidance are multifaceted, including the surgeon’s experience, zone of intervention, and alterations to normal anatomy [15]. In optimal scenarios, where operators are skilled and most patients undergo surgery for degenerative cervical spine disease, breach rates have been reported to be below 1.1% [16]. Conversely, various reports have documented rates of breach surpassing 33% in cervical pedicle screws inserted using fluoroscopic guidance [17]. These high breach rates may be attributed to the difficulties in interpreting two-dimensional images when assessing intricate three-dimensional structures. Despite these instances illustrating two extreme cases, cervical pedicle screw misplacement rates are estimated to be between 11 and 14% when using fluoroscopy [18,19]. The stages of navigation technology development are listed below in chronological order:

#### 3.1.1. Preoperative CT-Based Navigation

**Figure 2:**
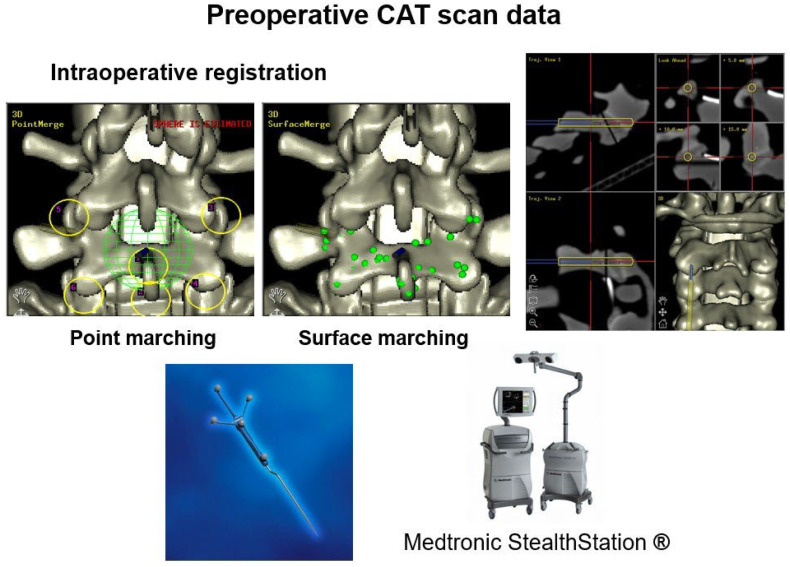
Images demonstrating preoperative CT scan data with different types of registration techniques in preoperative CT-based navigation.

Advancements in computer technology during the early 1990s facilitated the creation of frameless systems, which were initially utilized in brain tumor excisions [20]. The first stereotaxic systems used preoperative computed tomography images uploaded into an image processing computer. These images were subsequently aligned with the spinal anatomy of the individual in a surgical setting [21]. Various techniques have been employed for this registration, such as paired-point and CT fluoroscopy matching (Figure 2). In paired point matching, prominent structures are identified on the processed images, and their corresponding locations in the patient’s spine are marked and matched to the images. Conversely, CT fluoroscopy matching involves the use of fluoroscopy images in different directions to align with preoperatively obtained CT image data [22]. Regardless of the registration method applied, probe manipulation during surgical procedures allows for the adjustment of the corresponding three-dimensional anatomical imaging across multiple planes and depths, thereby facilitating pedicle screw placement.

Preoperative CT-based navigation has demonstrated early promise, with preliminary studies reporting enhanced accuracy over traditional methods in the cervical spine [23,24,25,26]. Nonetheless, several factors may impede the technical success and precise placement of pedicle screws, such as discrepancies in patient positioning [27]. Typically, patients undergo preoperative CT imaging in the supine position, whereas cervical pedicle screw insertion is generally performed in the prone position. These positional variations can modify intersegmental relationships by several millimeters, a factor that the navigation system does not consider [22]. The registration process is another critical source of error. In addition, intraoperative movement of the reference frame can cause registration errors with related pedicle screw misplacement. As a result, there remains a risk of critical transpedicular screw malpositioning in the cervical spine [28].

#### 3.1.2. Fluoroscopy-Based Navigation

**Figure 3:**
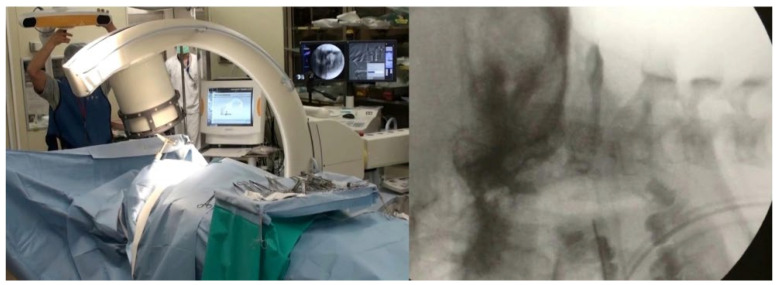
Fluoroscopy-based navigation settings.

In the late 1990s, the introduction of computer-aided image guidance using conventional C-arm imaging marked a significant advancement. This system has appealed to many surgeons, as most of them regularly employ fluoroscopic guidance for placing pedicle screws [11]. Known as “virtual fluoroscopy,” 2D fluoroscopy-based navigation employs intraoperative fluoroscopy to assist in hardware placement. Similar to preoperative CT-based systems, virtual fluoroscopy comprises an image workstation, a fixed dynamic reference array (DRA), and specialized surgical tools that can be tracked within the surgical environment (Figure 3). Instead of relying on a preoperative CT scan, several fluoroscopic projections are captured during surgery to create a computer-generated model of the surgical level. This modeling process is automated, thus avoiding the time-consuming registrations required by preoperative CT-based systems [22].

Fluoroscopic navigation enhances the precision of cervical pedicle screw insertion compared with traditional fluoroscopy [29]. However, virtual fluoroscopy has notable limitations compared with 3D navigation techniques. Primarily, it lacks the three-dimensional spatial feedback that 3D guidance systems provide, leading to a higher incidence of pedicle screw misplacement [29]. Additionally, image quality is heavily influenced by the imaging system used and can deteriorate with a larger patient size or bone demineralization [27]. Lastly, while virtual fluoroscopy has been shown to decrease fluoroscopic radiation exposure [30], it still necessitates some level of radiation exposure for the surgical staff [31].

#### 3.1.3. Intraoperative CT-Based Navigation

**Figure 4:**
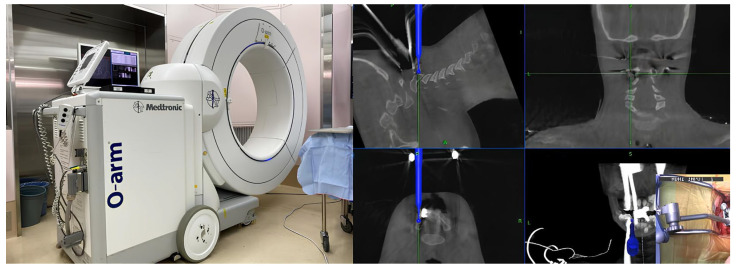
Intraoperative CT-based navigation.

In the early 2000s, the adoption of intraoperative CT-based navigation marked the advent of the most recent generation of sophisticated image-guidance systems for spinal surgery. The key innovation in intraoperative CT is the use of portable fluoroscopy units to capture CT-like datasets directly in the surgical field (Figure 4) [32]. Patients were scanned after obtaining the final position for surgery, so that intraoperative CT addresses positional discrepancies that can compromise the accuracy of preoperative CT systems. The quality of the CT images obtained in this manner is suitable for navigation because of the inherent contrast between the dense bony structures of the cervical spine and the relatively low attenuation of the surrounding soft tissues [33]. The electro-optic camera monitors and determines the DRA position during the initial CT scan, eliminating the need for a lengthy registration process. This technology allows repeated CT imaging if there is significant movement of the DRA or the patient during the procedure [32].

In the remainder of this manuscript, we explore the most relevant clinical evidence supporting the use of intraoperative three-dimensional image-guided navigation systems. They also addressed emerging challenges and proposed possible future directions for intraoperative image-guided interventions in cervical spine surgery.

### 3.2. Fields of Application

The incorporation of navigation systems into cervical spine surgery has significantly influenced these procedures. Initially, the focus of navigation systems was on ensuring the precision and safety of screw placement. However, with advancements in endoscopic techniques and the development of navigated tools such as osteotomes, drills, and burrs, the application of this technology has broadened to encompass nearly all areas of cervical spinal surgery. Research indicates that intraoperative navigation systems offer real-time 3D imaging, which enhances surgeons’ capabilities to navigate and perform complex anatomical operations while minimizing the risk of damage to nearby neurovascular structures.

#### 3.2.1. Cervical Spine Decompression

##### Anterior Approach

Although data on the use of this technology in anterior cervical surgery are scarce, its advantages have been noted in several studies. In the following sections, we briefly outline these circumstances.

1. Anterior Cervical Discectomy and Fusion

Although traditional anterior cervical discectomy and fusion (ACDF) utilizing fluoroscopy is a well-established technique with minimal complications [34], insufficient decompression of the cervical nerve root remains a prevalent issue. Given the close proximity of the vertebral artery to the anterior aspect of the cervical nerve root, often merely a millimeter apart, surgeons may be reluctant to perform extensive decompression in foraminal stenosis cases because of the risk of vascular injury. In scenarios where comprehensive decompression of the cervical nerve root is imperative, intraoperative navigation systems are exceedingly advantageous [35]. This technology can enable more precise and thorough decompression, particularly when distal structures are not readily visible in the surgical field, as in revision ACDF [36], or when severe bone degeneration obscures the natural bony landmarks [37]. Additionally, using intraoperative navigation in ACDF results in more accurate midline plate positioning compared to those placed with fluoroscopy guidance [38].

2. Minimally Invasive Anterior Cervical Spine Decompression

Endoscopic and microscopic spine surgeries are minimally invasive approaches that have been effectively adapted for anterior cervical spine interventions. By employing smaller incisions and tubular retractors, these techniques can reduce complications associated with soft tissue damage, as dysphagia and hoarseness [39,40,41]. The use of intraoperative three-dimensional navigation in these techniques enhances decompression and facilitates the planning of a smaller, more precise incision, thereby optimizing the position of the tubular retractor and its trajectory [40]. Two techniques of anterior endoscopic cervical discectomy have been introduced: the transdiscal and transcorporeal approaches. Both approaches were shown to be effective in selected cases suffering from cervical disc herniation with radiculopathy [39,40,42]. Nevertheless, the potential for degenerative disc damage, which may accelerate the reduction in intervertebral disc height and potentially lead to spontaneous bone fusion following a transdiscal approach, has increased the popularity of the transcorporeal approach [40,42]. Kim et al., in their 8-patients series, concluded that transcorporeal anterior cervical microforaminotomy facilitated by intraoperative navigation is a safe and effective alternative for the treatment of cervical radiculopathy that preserves the disc from iatrogenic damage [40].

The anterior endoscopic cervical discectomy combined with interbody fusion techniques can achieve further advantages in terms of indirect decompression of neural structures, maintenance of cervical lordosis, and restoration of intervertebral height [43]. One significant difficulty in employing this endoscopic techniques is the restricted visibility, which may limit the application of fusion implants [41]. The accurate placement of these implants could also be facilitated by the utilization of intraoperative navigation technology [38].

3. Ossification of the Posterior Longitudinal Ligament Resection

**Figure 5:**
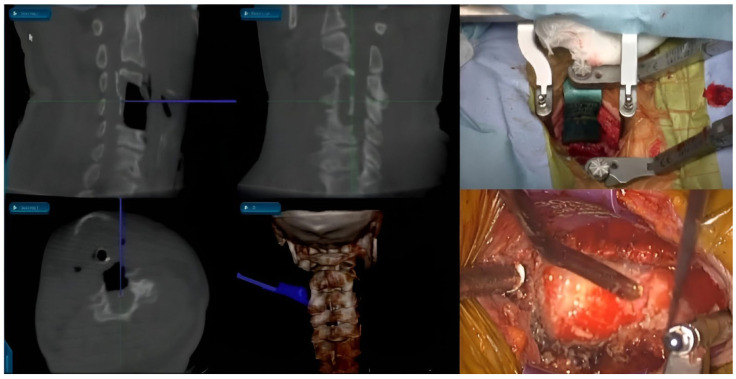
Intraoperative images showing the utilization of intraoperative navigation systems in OPLL resection.

Ossification of the posterior longitudinal ligament (OPLL) is a significant cause of cervical myelopathy and may necessitate anterior decompression in cases with profound spinal cord compression [44]. Although anterior decompression is effective for neurological recovery and restoring cervical lordosis, it has a high complication rate, such as dural tear and CSF leakage [45]. It is technically challenging, and long-term studies have shown poor improvement due to inadequate OPLL decompression [46]. Using intraoperative navigation can enable the surgeon to perform the anterior decompression more precisely by receiving live feedback for the location and the amount of decompression as demonstrated in Figure 5, improving the outcomes and reducing the rate of complications [9].

4. Endoscopic Endonasal Odontoidectomy

In the management of basilar invagination and odontoid abnormalities, the endonasal approach, in conjunction with occipitocervical fusion, has recently been adopted as an alternative to the traditional anterior transoral decompression [47,48]. Intraoperative navigation plays a vital role in this procedure because of the absence of reliable landmarks for nasopharyngeal fascia incision. Following this, navigation aids in assessing the necessary depth for drilling during bone removal [49]. Compared to transoral approaches, patients undergoing this procedure show earlier extubation times and experience less dysphagia postoperatively [50].

5. Corpectomy and Tumor Excision

Intraoperative navigation facilitates the precise determination of the corpus midline and the extent of bone removal during anterior cervical corpectomy. This technology enables the execution of a safe, extensive, and symmetrical corpectomy without jeopardizing the spinal cord or the vertebral arteries [36]. Furthermore, it enhances safety and confidence in performing multilevel corpectomies [36]. In cases involving tumors, intraoperative navigation systems are instrumental in achieving anterior resection of cervical tumors and effective decompression of the cervical cord without complications. These systems are crucial for accurately localizing the margins of soft tissue or osseous masses and vertebral arteries [51]. Preoperative MRI, coregistered and integrated with intraoperative CT, may function as an effective navigational tool during surgery to ensure the safe and thorough excision of complex spinal tumors [52].

##### Posterior Approach

1. Posterior Fossa Decompression

**Figure 6:**
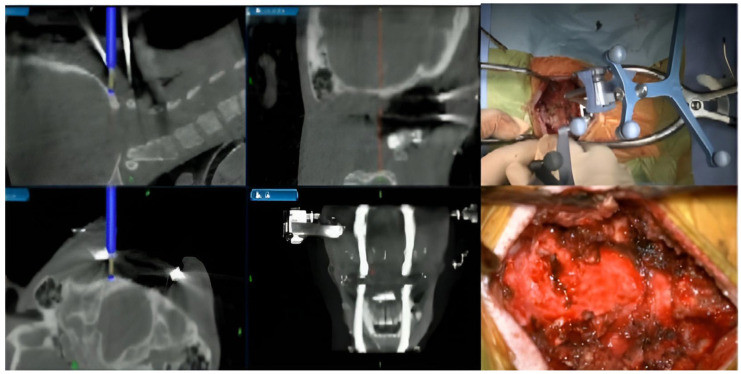
Intraoperative images showing the guidance of intraoperative navigation system in the craniotomy and upper cervical posterior arch resection.

Despite several surgical techniques for management of Chiari malformation are available, posterior fossa decompression is the most effective and safe procedure [53]. The efficacy of decompression mostly relies on the extent of bone resection; a limited craniotomy might result in inadequate decompression, whereas an extensive craniotomy might cause the descent of the cerebellum through the decompressed region [54]. Intraoperative navigation offers the option of visualization and prediction of the vital anatomic structures’ location in a relatively small working space and aids in the placement of the burr holes, allowing real-time intraoperative guidance (Figure 6). This is especially essential in conditions when congenital anomalies such as fused segments, hypoplasia of basioccipital bone, and platybasia coexist to ensure sufficient decompression and prevent iatrogenic complications in these challenging cases [4]. The navigation systems also facilitate C1 posterior arch resection and C2 laminectomy in severe tonsillar herniation [9]. Additionally, shorter surgical time was recorded in cases of posterior fossa decompression when intraoperative navigation were used [4].

2. Posterior Endoscopic Cervical Foraminotomy

Posterior endoscopic cervical foraminotomy is a minimally invasive technique for decompressing cervical nerve roots, minimizing soft tissue disruption, and consequently reducing intraoperative blood loss, postoperative pain, and dysfunction [55]. Nonetheless, the limited visualization inherent to endoscopy poses challenges in identifying anatomical structures and achieving sufficient decompression without excessive facet joint excision. Intraoperative navigation systems can mitigate these surgical challenges and enhance the accuracy and efficiency of posterior endoscopic cervical foraminotomy [55]. A notable advantage of the navigated approach is the ability to select an appropriate incision site and determine an optimal trajectory for dilator insertion, particularly in patients with short necks or at lower levels, which are difficult to verify using fluoroscopy [56]. Furthermore, direct bone resection can be precisely executed at the pathological site, especially for cervical foraminal stenosis, rather than creating a keyhole around the V-point, which is inconsistent owing to individual anatomical variations and patient positioning [57]. In a study involving 42 patients with foraminal stenosis or single-level intraforaminal disc herniation, Zhang et al. concluded that full endoscopic posterior foraminotomy assisted by intraoperative navigation is a safe and effective option for cervical radiculopathy, as navigation provides a real-time reference for adjusting the decompression field [57].

3. Cervical Unilateral Laminotomy for Bilateral Decompression

Patients with cervical spondylotic myelopathy, in the absence of instability, can be treated with minimally invasive cervical decompression using a unilateral laminotomy for bilateral decompression technique. In a 5-year cohort study, Minamide et al. found that cervical endoscopic unilateral laminotomy for a bilateral decompression approach provided comparable neurological recovery while preserving sagittal alignment, resulting in fewer axial symptoms than traditional cervical laminoplasty [58]. The intraoperative navigation systems were successfully utilized in these operations in incision site determination, localization of the lamino-facet junction, to obtain an appropriate trajectory of the tubular retractor for the contralateral decompression, and finally to confirm that bilateral decompression had been performed adequately [7].

#### 3.2.2. Cervical Spine Implant Placement

The anatomical structure of the cervical region poses unique surgical challenges, particularly at the upper cervical levels, owing to the presence of the spinal cord, nerve roots, and vertebral arteries, as well as the relatively limited bony areas available for instrumentation. Anatomically and biomechanically, the cervical spine can be categorized into the craniovertebral junction and the subaxial cervical spine.

##### Cranio-Vertebral Junction

Owing to the complex anatomical features and the distinct biomechanical properties of the cranio-vertebral junction, a high incidence of morbidity from both short- and long-term complications was traditionally deemed an inevitable outcome of surgery in this region. However, the introduction and implementation of technological advancements have enhanced the safety of these surgical procedures.

1. Occipital Condyle Screw

Occipital condyle screws offer several advantages, including a reduced lever arm length of the construct, the ability to insert longer screw lengths, and a lower profile construct that preserves a substantial occipital surface area for bone grafting and osseous fusion while mitigating complications related to wound healing [59]. Additionally, they can be integrated into traditional occipitocervical fixation systems for further augmentation. Technically, this type of fixation does not require rod bending, and because it aligns with the distal anchors (C1 and C2 screws), it facilitates the adaptation of rods into screw heads. Despite these advantages, the insertion of occipital screws can be technically challenging because of the relatively small area to navigate, the risks of neurovascular injuries, and the presence of condylar anatomic variations [10]. Le et al., in their case series of six patients involving the placement of 12 occipital condyle screws, demonstrated the efficacy and safety of intraoperative navigation, which could be regarded as a useful adjunct for the placement of these screws [60].

2. C1 Lateral Mass Instrumentation

Despite the rigid fixation, enhanced fusion rates, and decreased postoperative malalignment achieved with C1 lateral mass fixation compared to wiring techniques [61], the surgical approach requires a corridor surrounded by critical neurovascular structures [62]. Given that even a deviation of one or two millimeters in screw placement could result in devastating outcomes. Also, in the open traditional C1 screw placement, extensive exposure, encompassing the lateral mass and sulcus arteriosus, is necessary to identify the entry point. This procedure is often complicated by hemorrhage from the adjacent venous plexuses and, occasionally, from the vertebral arteries at unanticipated sites. Intraoperative navigation can be employed to ascertain the estimated screw entry points prior to soft tissue exposure, thereby necessitating only limited exposure around the entry point. This approach has led to a reduction in exploration time, minimal dissection, and decreased blood loss [63]. Many studies have demonstrated that intraoperative navigation has significantly improved the safe and accurate insertion of C1 lateral mass screws in traumatic [64], congenital [63], inflammatory [65], infectious, and neoplastic distorted anatomy [17], in which the anatomic landmarks are less reliable. These systems are also suitable for pediatric patients [63]. Although some studies have shown that navigated C1 screw placement accuracy is comparable to that of the fluoroscopic-guidance technique [66,67], intraoperative navigation allows the assessment of screw suitability, entry point, trajectory, and lateral mass dimensions [66].

3. C2 Odontoid Fracture Fixation

**Figure 7:**
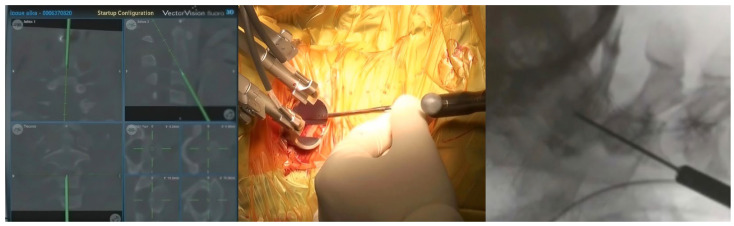
Identifying the entry point and trajectory of anterior odontoid screw using intraoperative navigation systems with its fluoroscopic confirmation.

Anterior fixation of unstable odontoid fractures offers the advantage of providing direct stabilization at the fracture site while the normal rotational movement of C1–2 is maintained, which is typically compromised with posterior C1–C2 fusion [68]. The accomplishment of these procedures necessitates appropriate imaging monitoring to confirm precise and safe screw insertion. Traditionally, this monitoring is achieved by C-arm, which cannot provide three-dimensional monitoring and results in excessive radiation exposure [30]. These limitations were expected to be overcome by intraoperative navigation (Figure 7). In addition to the reported cases [69,70,71], two cohorts utilizing two different intraoperative navigation systems (Iso-C^®^ and O-arm^®^) documented the safety and accuracy of these intraoperative adjuncts in the fixation of unstable odontoid fractures [72,73]. Other studies have compared the outcomes of anterior odontoid fixation utilizing traditional fluoroscopy image guidance with those achieved with intraoperative navigation assistance [74,75]. They concluded that, despite comparable clinical outcomes and complication rates, intraoperative navigation offers several advantages, including improved radiological fusion [74], reduced duration of surgery [74], decreased radiation exposure [74,75], and the capability to perform intraoperative three-dimensional imaging. This imaging allows for the confirmation of screw placement, enabling immediate correction in the event of misplacement, rather than necessitating a subsequent surgery.

4. C1–C2 Transarticular Screws

Among the various options for atlantoaxial fixation, posterior transarticular screws C1–C2 [76] offer the greatest biomechanical stability and fusion rates, with fusion rates potentially reaching as high as 98% [77]. Nevertheless, this method poses a high risk of neural and vascular injury due to anatomic location and patient variability. The risk associated with screw insertion is partly due to differences in the shape of the axis [78]. Radiological studies demonstrated that about one fourth of patients may not be suitable candidates for this type of fixation owing to abnormal vertebral artery course [79]. Uehara et al. in their 20-patient series demonstrated that intraoperative navigation reduces the risks of screw misplacement and consequent complications, with no complications reported, including vertebral artery tear [80]. Thereafter, Yang et al. compared intraoperative navigation with fluoroscopy-guided C1–C2 transarticular screws placement and illustrated the superiority of the intraoperative navigation over traditional fluoroscopy regarding accuracy, radiation exposure, and blood loss [81].

5. C2 Pars/Pedicle Screw Placement

**Figure 8:**
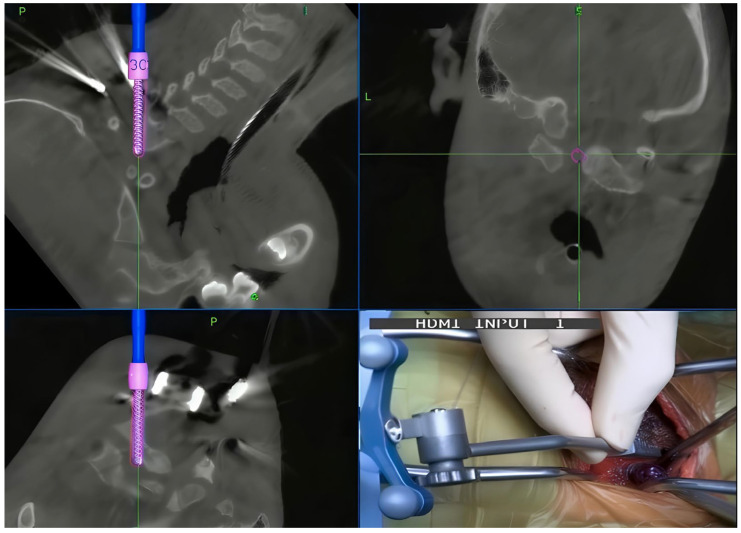
Images of C2 pedicle screw insertion using intraoperative navigation systems.

C2 pedicle and pars screws are frequently favored for C2 vertebra instrumentation due to their significant stabilization compared to translaminar screws [82] and the limited anatomic viability of C1–2 transarticular screws [79]. The proximity of the vertebral artery to the C2 pedicle and pars requires precise screw placement. The freehand screw placement requires more thorough exposure of the C2 arch to directly visualize the intended screw trajectory, and it is technically challenging, whereas navigation systems can facilitate accurate screw insertion with minimal dissection (Figure 8).

While no significant differences in complications were observed between patients undergoing the freehand and navigated techniques, initial studies suggested that the freehand technique demonstrated significantly greater accuracy than CT-based navigation for C2 pedicle and pars screw placement [10]. The potential sources of error in the navigated technique were attributed to the mobility of C2 relative to the reference frame, inaccuracies in registration, and the possibility of displaced reference frames occurring inadvertently during the surgery. As the navigated approach has been refined over the years, recent studies have indicated that the accuracy rate of the navigated group is comparable to that of the freehand group [83]. However, more recent reports proved the superiority of intraoperative navigated technique in cervical spine pedicle screw placement over conventional instrumentation [17,67], particularly for the C2 level with increased screw diameter/pedicle width ratio [67].

6. C2 Translaminar Screw Fixation

The primary challenges of translaminar screws fixation methods include achieving precise screw placement while avoiding these collisions and spinal canal encroachment. Intraoperative fluoroscopy does not significantly alleviate these technical challenges due to the inability to obtain an axial view. C2 translaminar screw placement can be substantially facilitated using intraoperative navigation by identifying the entry point, ensuring unobstructed crossing trajectories, and allowing insertion of maximal screw size without spinal canal breaching [84]. Studies have proven the safety, accuracy, and validity of intraoperative navigation in the C2 translaminar fixation with no complications reported, in addition to offering the possibility of intraoperatively sizing the screw to the patient’s anatomy [85,86].

##### Subaxial Cervical Spine

1. Subaxial Lateral Mass Screw

Owing to the proximity of crucial neurovascular structures and the narrow pedicle diameter in the subaxial cervical spine, Lateral mass screw fixation continues to be the predominant posterior fixation technique currently in use [87]. Despite its widespread application, it is not devoid of complications such as facet violation, lateral mass fractures and vertebral artery injury [88]. Nonetheless, intraoperative 3D navigation facilitates the planning of entry points, trajectories, and appropriate screw dimensions.

Arab et al. compared subaxial spine lateral mass screw insertion using CT-based navigation with freehand placement. They reported that the precision of navigated screw placement is significantly superior to freehand techniques [89]. These results were confirmed by another study using intraoperative navigation as a navigation system [17].

2. Subaxial (C3–C7) Pedicle Screw

Numerous studies have shown that cervical pedicle screws possess more desirable biomechanical properties than other fixation techniques [90]. Pedicle screws are reported to have a pull-out strength up to four times greater than lateral mass screws [91], which may allow for shorter instrumentation constructs. Despite the clear biomechanical advantages of cervical pedicle screws, the risk of complications from pedicle breaches should not be overlooked. The small size of the pedicle and the potential for breaches that could injure the cervical cord, nerve root, and vertebral artery have restricted its application. A recent meta-analysis comparing cervical pedicle screws with lateral mass screws found that the pedicle screw group had a significantly higher incidence of postoperative C5 palsy [92]. In a separate investigation, Abumi et al. reported the complication rate associated with the traditional placement of 712 cervical pedicle screws. The complications included two instances of nerve root injury attributable to screws, one nerve root injury resulting from foraminal stenosis, and one vertebral artery injury caused by a screw [92]. Critical pedicle breaches were observed in up to 27% of those placed under fluoroscopy. Even screws placed using preoperative computed tomography-based navigation systems exhibit complication rates of up to 18.7% [90]. The advanced application of intraoperative navigation systems has resulted in highly precise cervical spine screw placement, thereby achieving low complication rates (Table 1).

In a comparative study, Bertram et al. assessed the accuracy of cervical pedicle screw placement in 59 patients treated with intraoperative navigation versus 98 patients who underwent freehand placement [17]. They found that the precision of intraoperative navigated placement was significantly higher than that of freehand placement, with an initial accuracy of 93.28%, particularly in patients with degenerative cervical spine disorders.

3. Minimally Invasive Cervical Pedicle Screw (MICEPS)

**Figure 9:**
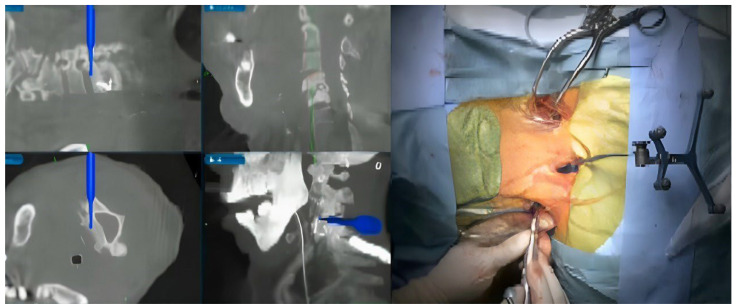
Intraoperative images showing the incisions and trajectories of MICEPS with aid of intraoperative navigation system.

In the cervical spine, the trajectory of pedicles is highly lateral to medial, with the C5 pedicle having an average sagittal angle of 46° [93]. This trajectory necessitates extensive dissection and a broader exposure for the conventional cervical pedicle screw placement. Such a wide exposure leads to considerable morbidity due to disruption of cervical muscles and the posterior ligamentous complex, resulting in associated postoperative neck pain. With advancements in intraoperative navigation and minimally invasive techniques, the minimally invasive insertion of these screws has become feasible (Figure 9), thereby mitigating the drawbacks associated with the traditional open approach.

Tokioka developed a method for subaxial screw insertion via an intramuscular posterolateral approach to minimize soft tissue damage, facilitate horizontal pedicle screw insertion, and prevent lateral misplacement of screws and vertebral artery injuries [94]. Initially, this technique was described for traumatic patients; subsequently, its indications were broadened to encompass cervical instability due to metastatic tumors, infectious conditions, and degenerative cervical spinal disorders [95,96]. In a comparative study evaluating patients undergoing subaxial cervical fixation with either conventional cervical pedicle screw or MICEPS technique fixation using intraoperative navigation, significantly superior clinical outcomes were reported in the MICEPS group in terms of screw placement accuracy and intraoperative bleeding [28].

## 4. Discussion

As with all emerging technologies, various forms of intraoperative navigation possess distinct advantages, yet they are not without disadvantages and challenges, thereby necessitating comprehensive investigation and balanced discourse. In this review, we aim to outline the benefits, drawbacks, and potential future developments of these systems in the following points.

### 4.1. Advantages

#### 4.1.1. Appropriate Level Localization

During surgical procedures, navigation systems enable surgeons to consistently verify the correct level without exertion or exposure to radiation by simply touching the navigated tools to the bony structures. This capability effectively prevents wrong-level surgery.

#### 4.1.2. Accurate and Safe Decompression and Fixation

The safety of intraoperative navigation systems and their increased accuracy in decompression and screw placement, particularly in the cervical spine, as demonstrated in the main body of this review, have been widely accepted. These advantages appear to be evident in both upper and subaxial cervical spines, when employed in high-risk surgical anatomy, even minor improvements in precision might justify the implementation of these systems.

#### 4.1.3. Live Feedback Provision

Spinal navigation systems offer surgeons uninterrupted three-dimensional visual feedback throughout surgical procedures. This technology enables surgeons to consistently verify the extent of decompression and the positioning of implants in relation to the spinal bony anatomy, as they receive real-time feedback during surgery [9].

#### 4.1.4. Intraoperative Screw Sizing

Intraoperative navigation systems provide significant advantages in determining the precise size and length of screws during cervical spine surgery. These systems facilitate accurate assessment of pedicle and lateral mass dimensions, allowing for customized screw selection based on individual anatomical variations, thereby ensuring optimal fixation strength and stability.

#### 4.1.5. Accurate Implant Placement in Complex Cervical Spine Conditions

Intraoperative navigation has significantly enhanced the safe and precise insertion of screws, especially in cases involving congenital abnormalities or anatomies altered by trauma, where conventional anatomical landmarks might be unreliable. This technology reduces the risk of injuring the vertebral artery or penetrating the cervical cord.

#### 4.1.6. Opportunity for Intraoperative CT Assessment

Intraoperative navigation systems offer the opportunity to repeat imaging to verify the accurate placement of the implants during surgery. In addition to ensuring accurate screw placement, the ability to correct any misplaced screws intraoperatively enhances the precision of navigated screw insertion, thereby reducing the likelihood of future interventions due to screw malposition [17].

#### 4.1.7. Facilitate Minimally Invasive Surgery

Spinal navigation systems facilitate the execution of minimally invasive surgeries on the cervical spine. These systems assist spine surgeons in accurately determining the entry point and trajectory for cervical pedicle screws, even in the absence of extensive exposure of anatomical landmarks and surrounding soft tissue, thereby supporting a minimally invasive surgical approach [97].

#### 4.1.8. Decrease Blood Loss

Using navigation results in considerably reduced blood loss. This reduction can be attributed to the minimally invasive technique, as navigation allows the surgeon to directly access the entry point and perform a smoother dissection, particularly around the dense venous plexus in the upper cervical spine [67].

#### 4.1.9. Motion-Preserving Approach

Intraoperative navigation can be employed to preserve motion segments during fusion surgeries, allowing for the stabilization of the cervical spine by fusing fewer mobile segments. For instance, atlantoaxial fusion, which results in a postoperative reduction in cervical range of motion, is typically indicated for unstable atlas fractures. However, recent studies have shown that atlas osteosynthesis with intraoperative navigation offers a safe and effective method for maintaining motion while treating unstable atlas fractures [67]. Furthermore, evidence suggests that cervical pedicle screws, whose placement is significantly facilitated by intraoperative navigation systems, offer superior biomechanical stability and greater pullout strength compared with alternative fixation methods. This advantage may permit the use of shorter instrumentation constructs [98].

#### 4.1.10. Shorter Learning Curve

Mastery of precise dorsal cervical screw insertion is a complex process, with accuracy largely dependent on the surgeon’s experience [99]. Nonetheless, the learning curve for navigated cervical pedicle placement may be relatively safer and shorter, assuming sufficient training and familiarity with the navigation system [100].

#### 4.1.11. Reduced Radiation Exposure to the Surgical Staff

Computed tomography (CT)-based intraoperative navigation systems require an initial registration scan before the commencement of a surgical procedure. During this scan, all members of the surgical team and staff vacate the operating room and remain shielded behind lead barriers in substerile areas, thereby effectively eliminating radiation exposure for all individuals, except the patient.

### 4.2. Disadvantages

#### 4.2.1. Technical Difficulties

Owing to the high costs associated with the acquisition and maintenance of navigation systems, there is ongoing debate regarding whether these expenses are justified by the potential benefits [101]. The intricate setup required for these systems prior to their use in cervical spine surgeries might partially explain why surgeons do not consistently achieve enhanced accuracy with intraoperative navigation [102]. The mobility of the spine, coupled with the restricted working space, necessitates the meticulous management of the dynamic reference frame (DRF). It is imperative for surgeons to register the DRF immediately before utilizing the navigation system, optimally following the exposure of the vertebral bony surface. This practice minimizes the risk of frame shifting relative to the vertebrae during the surgical procedure. Furthermore, the DRF should be positioned in close proximity to the target vertebrae to ensure optimal registration and navigational accuracy. In procedures involving the upper cervical levels, the positioning of the DRF may present fewer challenges because it can be secured outside the surgical field on a rigid Mayfield holder. Conversely, for the subaxial cervical spine, the DRF must be retained within the surgical field and affixed to the spinous processes of vertebrae, such as C2 or T1. The proximity of the DRF during navigation requires surgeons to be vigilant to avoid unnecessary contact and movements. If accidentally disturbed, the accuracy of the navigation system may be compromised [103]. Furthermore, after placing each pedicle screw, the surgeons should verify that the navigation system continues to accurately track the vertebrae. Each screw placement can cause relative movement between individual vertebrae and, consequently, relative movement toward the DRF [104].

To prevent inaccuracies arising from discrepancies between the trajectories of the screwdriver and screw, it is advisable to intermittently release the pressure from the screwdriver to verify the actual trajectory of the screw. Surgeons must possess a thorough understanding of the navigation system owing to its complex configuration. Even a minor oversight can rapidly compromise the accuracy of the navigation system, thereby affecting the precise placement of the cervical pedicle screws.

#### 4.2.2. Increased Radiation Exposure to the Patients

Research involving cadaveric models has demonstrated that the application of intraoperative navigation decreases the surgeon’s radiation exposure compared to the freehand fluoroscopic technique; however, it results in increased radiation exposure to the cadaver [105]. This observation has been corroborated in clinical settings, where the radiation dose associated with navigation is approximately three times greater than that associated with fluoroscopy [106]. Furthermore, another study reported that the radiation dose administered to patients during intraoperative CT was significantly higher than that received when the surgical team was present in the operating room (OR). On average, the radiation exposure to the patient was 8.74 times greater than that when the surgical team was inside the OR. This disparity in radiation exposure was particularly pronounced during cervical and cervicothoracic spine procedures, where the total radiation dose to the patient could be as much as 17.42 times greater than when the surgical team was present in the OR [107].

#### 4.2.3. Longer Operative Time

Previous studies have indicated that the use of intraoperative navigation in posterior instrumentation of the cervical spine, particularly at the craniovertebral junction, is associated with extended operative times owing to prolonged preparation and setup. This extension of operative duration may be correlated with an increased rate of postoperative complications [66]. It is important to note that the introduction of any novel technique is accompanied by a learning curve, which initially results in longer operative durations. However, as proficiency with the technique is developed over time, the duration of surgery tends to decrease, eventually aligning with that of conventional non-navigated approaches [64,108]. Broader navigation literature demonstrates that both setup and overall operative time decrease significantly with surgeon and team experience, often plateauing after approximately 30 cases as proficiency is achieved, furthermore modern 3D navigation can reduce the need for repeated fluoroscopic scans and interruptions, further narrowing the operational efficiency gap with conventional techniques [109].

#### 4.2.4. Cost and Availability

Reports suggest that intraoperative navigation may not be as cost-effective as traditional spinal fusion during the initial hospitalization. A thorough cost-effectiveness analysis incorporating long-term outcome data is essential to address this issue [110]. Conversely, available cost-effectiveness evidence suggests that intraoperative navigation in cervical spine surgery is most advantageous in clinically complex scenarios involving severe deformity, revision surgery, or high-risk anatomy, where improved screw placement accuracy and reduced complication or reoperation rates offset the added equipment and operative time costs. Thus, employing image-guided navigation in high-volume centers that focus on complex cases offers a cost advantage [111]. The substantial costs associated with its implementation contribute to its limited adoption. Moreover, affordability presents a significant challenge, as many hospitals, particularly those in low- and middle-income countries, cannot afford these systems.

### 4.3. Future Direction

Intraoperative navigation in cervical spine surgery is poised for advancement through the integration of augmented reality (AR), robotics, and other state of the art technologies designed to address existing challenges in precision, radiation exposure, workflow efficiency, and cost-effectiveness. Preliminary clinical and experimental findings underscore the potential and nascent stages of these innovations in the field. For example, AR-enhanced navigation systems have demonstrated improved accuracy in pedicle screw placement in randomized and pilot clinical trials compared to traditional methods, offering real-time visualization that reduces the need for frequent fluoroscopy and may enhance workflow by minimizing distractions from the surgical field [112,113]. A recent clinical feasibility study on AR navigation for atlantoaxial pedicle screw fixation reported consistently safe screw placement with high user satisfaction, indicating the applicability of this technology even for anatomically complex cervical procedures [114]. Although comprehensive cost–benefit analyses remain necessary, systematic reviews suggest that AR can maintain or enhance workflow efficiency and potentially reduce radiation exposure for both patients and staff [112].

Similarly, robot-assisted navigation systems, particularly when combined with intraoperative imaging such as C-arm or O-arm scans, have demonstrated high accuracy in cervical pedicle screw insertion in cadaveric and early clinical studies, potentially surpassing conventional navigation in terms of precision [115]. These robotic systems promise to reduce variability from manual techniques, shorten learning curves for complex procedures, and further decrease reliance on ionizing radiation when integrated with advanced imaging and registration algorithms. Theoretically, the incorporation of artificial intelligence (AI) and machine learning, such as marker-less segmentation, automated trajectory prediction, and dynamic error correction, could enhance registration speed and accuracy, reduce setup and intraoperative time, and lower cumulative radiation exposure by optimizing imaging requirements [116]. However, empirical data remain limited, particularly regarding long-term clinical outcomes and cost-effectiveness in the cervical spine.

Future research should focus on prospective multicenter trials to assess how these technologies enhance surgical precision, reduce operative time and radiation exposure, and impact overall costs compared with established navigation methods. Additionally, studies that integrate AR, robotics, and AI into unified intraoperative platforms could elucidate how combined systems might streamline workflows and make advanced navigation more accessible in routine cervical spine practice.

## 5. Conclusions

Intraoperative navigation plays a crucial role in enhancing the safety and effectiveness of cervical spine surgery. Although its initial application was limited, it has now expanded to encompass most surgeries involving the cranio-vertebral junction and subaxial cervical spine. A recent trend supports the routine use of these systems in all cervical spine surgeries. However, navigation should primarily function as a guide to assist the surgeon rather than being solely relied upon without confirmation from the anatomical landmarks. Regular accuracy checks are vital for ensuring precise navigation and mapping. Continued research and development in this field are essential for creating highly integrated and intelligent navigation systems that optimize surgical outcomes. Ultimately, establishing the long-term clinical and economic benefits of modern navigation systems requires further high-quality level 1 comparative research to confirm their cost-effectiveness relative to conventional techniques.

## Figures and Tables

**Table 1:** Subaxial Cervical Pedicle Screw Placement: Navigation vs. Non-Navigation.

Parameter	Navigation-Assisted	Non-Navigation (Freehand/Fluoroscopy)
Typical accuracy rate	90–98% pedicles fully contained	70–90% pedicles fully contained
Critical pedicle breach (>2 mm)	1–5%	8–27%
Medial breach risk (spinal canal)	Low (≈1–3%)	Higher (≈5–15%)
Lateral breach risk (vertebral artery)	Very low (≤1–2%)	Higher (≈3–10%)
Vertebral artery injury	Rare (<1%)	Rare but higher than navigation (≈1–3%)
Neurologic complication	<1–2%	1–5%
Revision surgery for malposition	<1–2%	3–10%
Radiation exposure to surgeon	Minimal	Higher (fluoroscopy-dependent)
Operative time	Slightly longer initially; comparable with experience	Shorter initially
Learning curve	Moderate; improved consistency	Steep; highly surgeon-dependent
Utility in deformity/trauma	High (distorted anatomy)	Limited/higher risk
Cost and equipment	Higher	Lower

## Data Availability

The data presented in this study are available in the article.

## Abbreviations

The following abbreviations are used in this manuscript:

CT	Computerized Tomography
PRISMA	Preferred Reporting Items for Systematic Reviews and Meta-Analyses
MeSH	Medical Subject Headings
2D	Two Dimensional
3D	Three Dimensional
DRA	Dynamic Reference Array
ACDF	Anterior Cervical Discectomy and Fusion
OPLL	Ossification of Posterior Longitudinal Ligament
CSF	Cerebrospinal fluid
MRI	Magnetic Resonance Imaging
MICEPS	Minimally Invasive Cervical Pedicle Screw
AR	Augmented Reality
AI	Artificial Intelligence
